# A naturally-occurring phenomenon of flower color change during flower development in *Xanthoceras sorbifolium*

**DOI:** 10.3389/fpls.2022.1072185

**Published:** 2022-11-15

**Authors:** Yanan Lu, Hanhui Wang, Zhi Liu, Tianxu Zhang, Zongjian Li, Li Cao, Siyao Wu, Yueying Liu, Song Yu, Qingzhu Zhang, Zhimin Zheng

**Affiliations:** ^1^ State Key Laboratory of Tree Genetics and Breeding, College of Forestry, Northeast Forestry University, Harbin, China; ^2^ College of Life Science, Northeast Forestry University, Harbin, China

**Keywords:** anthocyanin, MYB113, DNA methylation, *Xanthoceras sorbifolium*, flower development

## Abstract

*Xanthoceras sorbifolium* (yellowhorn) is originated in China and is a unique tree in northern China. Yellowhorn is very popular because of the gradual color change of its flower at different flower developmental stages. During flowering development, the color at the base of yellowhorn flower petals gradually changes from yellow to purple. The mechanism of this miraculous phenomenon is still unclear. Here we show that anthocyanin accumulation during flowering development is the main reason for this color change. RT-PCR results show that the expression level of a variety of anthocyanin biosynthesis genes changes in different flower developmental stages. Realtime results show that the expression changes of these anthocyanin biosynthesis genes are positively regulated by a cluster of *R2R3-MYB* transcription factor genes, *XsMYB113s*. Furthermore, the DNA methylation analysis showed that CHH methylation status on the transposon element near the *XsMYB113-1* influence its transcript level during flowering development. Our results suggest that dynamic epigenetic regulation of the *XsMYB113-1* leads to the accumulation of anthocyanins during yellowhorn flower color change. These findings reemphasize the role of epigenetic regulation in flower development and provide a foundation for further studies of epigenetic regulation in long-lived woody perennials.

## Introduction

The flower color of yellowhorn is various, bright and colorful. Yellowhorn is a woody oilseed-bearing shrub endemic to Northern China with both agricultural and horticultural commercial significance. This member of the Sapindaceae family is commonly grown where soil and water conservation are essential due to its ability to thrive on barren, saline, or alkaline soil. Because of the beautiful colors and form of its flowers, *X. sorbifolium* is used as an ornamental plant. Several varieties of *X. sorbifolium* have been established based on their petal color.

The petals of white-flowered trees change color from yellow to purple at their flower base during development, while the rest of each petal remains white. In contrast, entire petals change color from yellow to red in red-flowered trees, but turn from yellow to red and eventually to purple in the flowers of purple-flowered trees. The mechanisms controlling color of *X. sorbifolium* flower petals have not yet been adequately studied.

Different insect pollinators can select for certain flower colors (Campbell et al., 2010). Plants with different colors will attract different kinds of insects to visit, so as to achieve the purpose of pollination. Yellowhorn displays different flower colors at different flower developmental stages, which may attract different kinds of pollinators. *Xanthoceras sorbifolium* is a woody oilseed-bearing tree, but its yield is not high. Studying the gradual change of the yellowhorn flower color may have positive significance for improving the yield of yellowhorn through insect pollination.

The formation of different flower colors is often related to the type and content of anthocyanins in the petals. The anthocyanin biosynthesis pathway (ABP) genes are responsible for the synthesis of plant anthocyanins, and the anthocyanin synthesis pathway is a part of the flavonoid metabolic pathway (Holton and Cornish, 1995). The research on the synthetic pathway of anthocyanin started earlier, and the research on the synthetic pathway of plant anthocyanin has been relatively clear. Many regulators of anthocyanin biosynthesis have been identified in different species. Among them, MYB, basic helix-loop-helix (bHLH) and WD40 repeat (WDR) proteins are clearly studied. They can combine with each other and form a complex to participate in the regulation of anthocyanin (Koes et al., 2005; Feller et al., 2011; Hichri et al., 2011).

A cotton R2R3-MYB113 transcription factor was reported to directly mediate the formation of purple spot at the inner base of cotton petal. Further research showed that the purple spot was related to the frequency of honeybees visiting flowers, which was beneficial to improve cotton seed yield (Abid et al., 2022). Overexpression of *AtMYB113* in Arabidopsis resulted in a distinct pigmentation phenotype in plants, and the accumulation of pigments was found to be dependent on TTG1 and bHLH (Gonzalez et al., 2008). In potato, overexpression of *StMYB113* can significantly increase anthocyanin synthesis in tobacco leaves, and it was further found that StbHLH1 and StJAF13a are co-regulators of anthocyanin synthesis (Liu et al., 2016).

Studies have shown that DNA methylation is related to pigmentation. For example, the level of DNA methylation in the *MdMYB1* promoter region in red-striped apples is much higher than that in red-skinned apples. And the higher the level of DNA methylation, the lower the anthocyanin content in the apple skin, and the lighter the skin color (Ma et al., 2018). A similar phenomenon also appeared in pears. The level of DNA methylation in the *PcMYB10* promoter region in pears with green fruit epidermis was significantly higher than that in pears with red epidermis, and the function of this gene was proved to regulate anthocyanin synthesis in pears. High levels of DNA methylation repressed the expression of the *PcMYB10* gene, thereby reducing the expression level of the *UFGT* gene, resulting in differences in anthocyanin accumulation in different cultivars (Wang et al., 2013). In tomato, hypermethylation of DNA in the promoter region of the *CNR* gene results in inhibition of fruit ripening (Zhong et al., 2013). In apple, the red skin phenotype is associated with the insertion of a long terminal repeat (LTR) retrotransposon upstream of *MdMYB1* that activates transcription for anthocyanin biosynthesis. Thus, variation in methylation of this LTR transposable element leads to different apple skin colors (Zhang et al., 2019).

Epigenetic regulation of gene expression occurs during many significant biological processes (Zhang and Zhu, 2012). DNA methylation, a stable yet reversible epigenetic state, plays important roles in transposon silencing, genomic imprinting, and maintaining genomic stability (Zhang et al., 2018). Methylation occurs at the fifth position on cytosine residues (5mC) in DNA and has been found in plants in three sequence contexts in plants: CG, CHG, and CHH, where H stands for either A, C, or T (Lister et al., 2008). CHH methylation is particularly important due to its ability to regulate gene expression.

The establishment and maintenance of CHH methylation is controlled by the RNA directed DNA methylation (RdDM) pathway (Zhang and Zhu, 2011; Matzke and Mosher, 2014; Wendte and Pikaard, 2017). Then either DRM2 or CMT2 (CHROMOMETHYLASE 2) installs methylation *de novo* at CHH, depending on its genomic location. DRM2 installs CHH methylation at RdDM target sites or at the ends of long transposons, usually in euchromatic regions, while CMT2 installs CHH methylation in heterochromatic regions in a manner dependent upon DDM1 (Decrease DNA Methylation 1) (Zemach et al., 2013). The newly discovered CMT2-to-RdDM pathway can inhibit transposition when DDM1 function is deficient (He et al., 2021). Therefore, RdDM is important for epigenetic silencing of some transgenes and endogenous repeats (Zheng et al., 2015).

In the present study, we analyzed the anthocyanin components in the petals of yellowhorn flowers during four developmental stages starting from the bud (S1), to newly opened flowers with yellow coloration at the inner base of the petals (S2), to opened flowers with orange at the inner base of the petals (S3), to fully open flowers in which the color of the inner petal base becomes redder (S4). The accumulation of cyanidin and other anthocyanins is likely to be the main cause of the color changes observed in yellowhorn flowers.

We identified a cluster of *XsMYB113*/TEs associated with the accumulation of anthocyanins in the petals of yellowhorn. The results of DNA methylation sequencing revealed that the expression level of *XsMYB113-1* gene was regulated by DNA methylation on the upstream transposon. Therefore, as observed for development and ripening in other horticultural species, DNA methylation could also have important functions in the development and color change of yellowhorn flowers. Heterologous overexpression of *XsMYB113* genes in tobacco demonstrated that they all positively regulate anthocyanin synthesis.

## Materials and methods

### Plant materials

Yellowhorn trees analyzed for the present study were sampled from the Kundu Economic Forest Farm (E119°96’ N44°24’, Ar Horqin Banner, Chifeng City in Inner Mongolia, China) and the Northeast Forestry University campus (E126°64’ N45°72’, Harbin City in Heilongjiang Province, China), where they grew under natural conditions. Whole flowers from four developmental stages (S1 through S4) were used as experimental material. All samples were collected in three biological replicates from a minimum of three different individual trees. All flower samples were immediately frozen in liquid nitrogen and stored at -80°C until use.

For transgenic analysis of the functions of *XsMYB113* genes in this study, we used the wild-type tobacco (*Nicotiana tabacum*) variety Yun87 that was developed from a cross between the female parent Yunyan 2 and the male parent K326 from the Yunnan Academy of Tobacco Agricultural Sciences, followed by pedigree selection. We grew wild-type and transgenic tobacco lines at 22°C on a 3:1 mixture of soil:vermiculite under a photoperiod of 12 h light:12 h dark and then plants were transferred to a glasshouse to grow under natural light. Fresh leaves and flowers of transgenic or WT tobacco plants were collected for extracting DNA and RNA to identify their genotypes and analyze transcript expression.

## Methods

### Metabolite profiling

Anthocyanins in whole yellowhorn flowers from four developmental stages (S1 through S4) were identified and quantified at the Shanghai Lingen Biological Technology Co., Ltd. using a liquid chromatography-electrospray ionization-tandem mass spectrometer (LC-ESI-MS/MS) system [HPLC, CBM20A system (Shimadzu, Kyoto, Japan); MS, 4500 QTrap (Applied Biosystems, Waltham, US)]. At each stage, all samples were collected in three biological replicates from a minimum of three different individual trees.

### Extraction and quality evaluation of total RNA

All samples were lyophilized and then powdered in LN_2,_ weighed (50 mg), and total RNA was isolated using the RNAprep Pure Plant Kit from Tiangen Biotech (Beijing, China). Using a NanoDrop ND–2000 (Thermo Fisher Scientific, Waltham, MA, USA), the quality and quantity of each sample was measured by taking its absorbances at 260/280 and 260/230 nm, based on an extinction coefficient of 7,700. RNA integrity was assessed by electrophoresis of RNA samples on denaturing formaldehyde gels.

### qRT-PCR assays

Total RNA samples (1 µg) from yellowhorn and tobacco were transcribed into cDNA using the PrimeScript^TM^ RT Reagent Kit with gDNA Eraser (Takara Biomedical Technology Co., Ltd., Beijing, China) following the manufacturer’s protocol. In *X. sorbifolium*, we used the *XsACTIN* gene as an internal control. In tobacco, we used the *NtEF1α* gene as an internal control, and the sequence of the primer used for detecting its expression was published previously (Beinecke et al., 2018). All primers used for qRT-PCR are shown in **Supplementary Table S4**.

### Phylogenetic analysis

We performed blastp (https://blast.ncbi.nlm.nih.gov/Blast.cgi) comparisons to identify homologs of genes encoding anthocyanin biosynthesis-related MYB transcription factors and DNA methyltransferase. The *X. sorbifolium* protein sequences with similarity >60% to these homologs were chosen for further study. We constructed a phylogenetic tree using Maximum Likelihood (Gourieroux et al., 1984) (ML) in MEGA 7 (Kumar et al., 2016) on our local server. We have listed the GenBank accession numbers of these sequences in **Supplementary Tables**.

### Plasmid construction and generation of transgenic plants

The CDSs of the *XsMYB113-1, XsMYB113-2, XsMYB113-3*, *and XsMYB113-4* genes from *X. sorbifolium* trees were amplified from cDNA using Q5® High-Fidelity DNA Polymerase (New England Biolabs, Ipswich, MA, USA) and then cloned into pEASY®-Blunt Zero vectors (TransGen Biotech). We sequenced all clones, aligned the sequences with the CDSs in the *X. sorbifolium* reference genome, and found no sequence differences. We then used the Trelief™ SoSoo Cloning Kit Ver.2 (Tsingke Biotech, Harbin, China) to clone the full-length cDNAs of four genes into pBI-121 vectors (EK-Bioscience, Shanghai, China) under control of the CaMV 35S promoter to create *35S::XsMYB113-1, 35S::XsMYB113-2, 35S::XsMYB113-3*, and *35S::XsMYB113-4* constructs. All constructs used in the present study were confirmed by Sanger sequencing at Tsingke Biotech. These overexpression vectors were then transformed into tobacco (Yun87) using the leaf disc method (Horsch et al., 1985) and selection of transgenic shoots on MS (Murashige and Skoog) medium (PhytoTechnology, Lenexa, KS, USA) with kanamycin. Kanamycin-resistant shoots were removed for induction of roots on MS medium (PhytoTechnology, Lenexa, KS) containing 1-naphthylacetic acid (NAA). Twenty transgenic lines per construct were obtained. We confirmed putative transgenic lines using PCR and qRT-PCR with transgene-specific primers.

### Anthocyanin quantification

We measured anthocyanin contents as described previously (Deikman and Hammer, 1995) with some modifications. Samples of leaves and flowers were ground into powder in LN_2_ (liquid nitrogen). Roughly 0.5 g of powder was suspended in 2 ml of 1% (v/v) HCl in methanol and chilled overnight at 4°C. After centrifuging the samples for 20 min at 12,000×g absorbances of the supernatant at 535 and 650 nm were taken using a spectrophotometer (INESA, Shanghai, China. We have presented anthocyanin contents as (A535-A650)/g FW (fresh weight), based on an extinction coefficient of 4.62×10^6^.

### Bisulfite sequencing

BSP-PCR assays were performed as described in a previously published article (Telias et al., 2011). We extracted genomic DNA from *X. sorbifolium* flowers using the DNeasy Plant Maxi Kit (Qiagen, Dusseldorf, Germany). 500 ng genomic DNA was treated by EpiTect Bisulfite Kit (Qiagen, Dusseldorf, Germany). The product was used as a template to amplify the *RTE* transposon fragments using the Q5® High-Fidelity DNA Polymerase (New England Biolabs, Ipswich, MA, USA) and cloned into pEASY®-Blunt Zero vectors (TransGen Biotech), and then sequenced. 30 independent clones of PCR were were analyzed by Kismeth software (Gruntman et al., 2008).

### Analysis and classification of the transposon near the *XsMYB113* genes

Repbase (Kohany et al., 2006) (https://www.girinst.org/censor/index.php) was used to identify transposon sequences near the *XsMYB113* genes. Transposon sequences were annotated using RepeatMasker (http://repeatmasker.org).

## Results

### The change in yellowhorn flower color is due to the accumulation of anthocyanins

**Figure 1:**
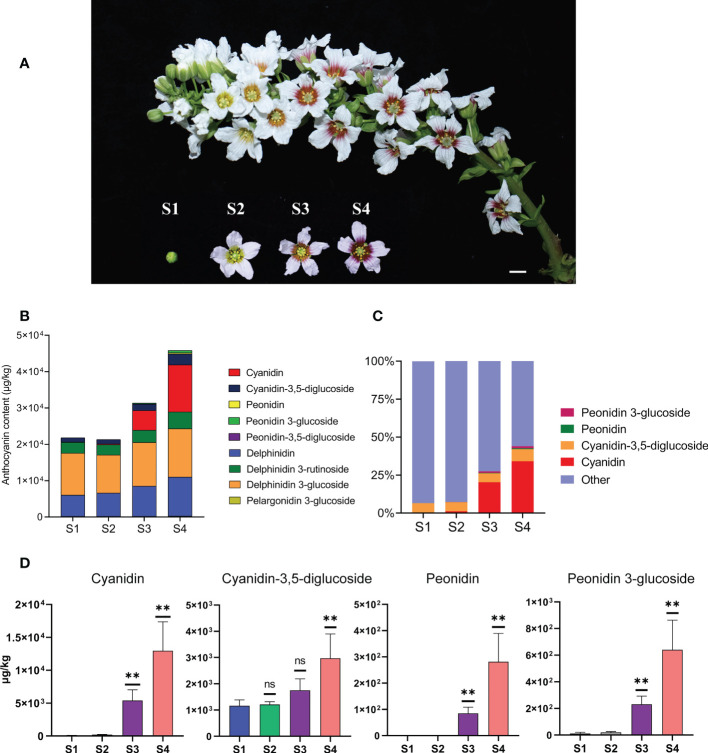
The content of anthocyanins was constantly changing during the process of flower development and flower color change in *X. sorbifolium.* **(A)** Flowers on the same inflorescence were divided into four developmental stages according to the color change showing at the base of petals. S1 (stage1, closed green buds, length < 1 cm), S2 (stage 2, opening flowers with yellow at the base of the petals, length 1–2 cm), S3 (stage 3, open flowers with orange at the base of the petals, length 1–2 cm), S4 (stage 4, open flowers with purple-red at the base of the petals, length 1–2 cm). Bar = 1 cm. **(B)** Nine kinds of anthocyanins were identified and quantified from the yellowhorn petals by targeted metabolism at S1 to S4. The data are the mean ± SD from three biological replicates. SD, standard deviation. **(C)** The proportion of the four anthocyanins (Cyanidin, Cyanidin-3,5-diglucoside, Peonidin, Peonidin 3-glucoside) with the most obvious accumulation in the total amount of anthocyanins at each developmental stage. Other represents the sum of the total content of the other 5 kinds of anthocyanins, including Peonidin-3,5-diglucoside, Delphinidin, Delphinidin 3-glucoside, Delphinidin 3-rutinoside and Pelargonidin 3-glucoside. The sum of total anthocyanins of different species in each developmental stage (S1 to S4) was normalized to 100%. **(D)** The four anthocyanins with the most obvious changes in content during yellowhorn flower color change, including Cyanidin, Cyanidin-3,5-diglucoside, Peonidin, Peonidin 3-glucoside. *P* values were calculated based on two-sided *t* test (**P < 0.01; ns: not significant).

Color change in yellowhorn flowers can be divided into four developmental stages (from S1 to S4). In the first stage (S1), the flower bud has not yet opened. After the flower buds have opened at S2, the flowers develop yellow color at the inner base of the petals and the outer petals remain white. At S3, the fully opened flowers are orange at the inner base of the petals and the outer parts petals remain white. At S4, the flowers are fully open and show purple color at the inner base of the petals and white outer petal parts (**Figure 1A**). We collected complete flower structures from S1 to S4 for metabolic characterization.

Individual metabolites were analyzed and 27 metabolites were identified, including 9 anthocyanins, 7 flavones, 3 flavanols, 1 flavonol, 7 organic acids (**Figure 1B**; **Supplementary Figure S1**). Firstly, we focused on analyzing the relationship between the content changes of 9 kinds of anthocyanins and the yellowhorn flower color change. It was found that five anthocyanins (Peonidin-3,5-diglucoside, Delphinidin, Delphinidin 3-glucoside, Delphinidin 3-rutinoside and Pelargonidin 3-glucoside) were synthesized at S1, and the content of each component was almost the same as that at S2. The total content of 5 anthocyanins increased through S3 to S4, but the proportion in total anthocyanins decreased (**Figures 1B, C**).

In all cases, the major anthocyanin detected was cyanidin (magenta), which increased about 213 times from 60.7 μg/kg at S1 to 12,953 μg/kg at S4, with significant difference (*P* < 0.01). The next most abundant anthocyanin, cyanidin-3,5-diglucoside (red-purple), increased about 2.6 times from 1162 μg/kg at S1 to 2973 μg/kg at S4, with significant difference (*P* < 0.05). Next, the content of peonidin (purple/blue) increased about 281 times from 0 μg/kg at S1 to 281 μg/kg at S4, with significant difference (*P* < 0.01). The content of peonidin 3-glucoside (dark red to purple) increased nearly 57 times from 11 μg/kg at S1 to 640 μg/kg at S4, with significant difference (*P* < 0.01) (**Figure 1D**). The proportion of the four anthocyanins in the total anthocyanin content in each developmental stage increased continuously with the flower development process, especially at S3 and S4, which was consistent with the stages when the flower color changed most obviously (**Figure 1C**). The remaining 18 metabolites were not as significant as the changes in anthocyanin content. These results indicated that the yellowhorn flower color change is closely related to the accumulation of anthocyanins.

### Increased expression of ABP pathway genes leads to accumulation of anthocyanins

**Figure 2:**
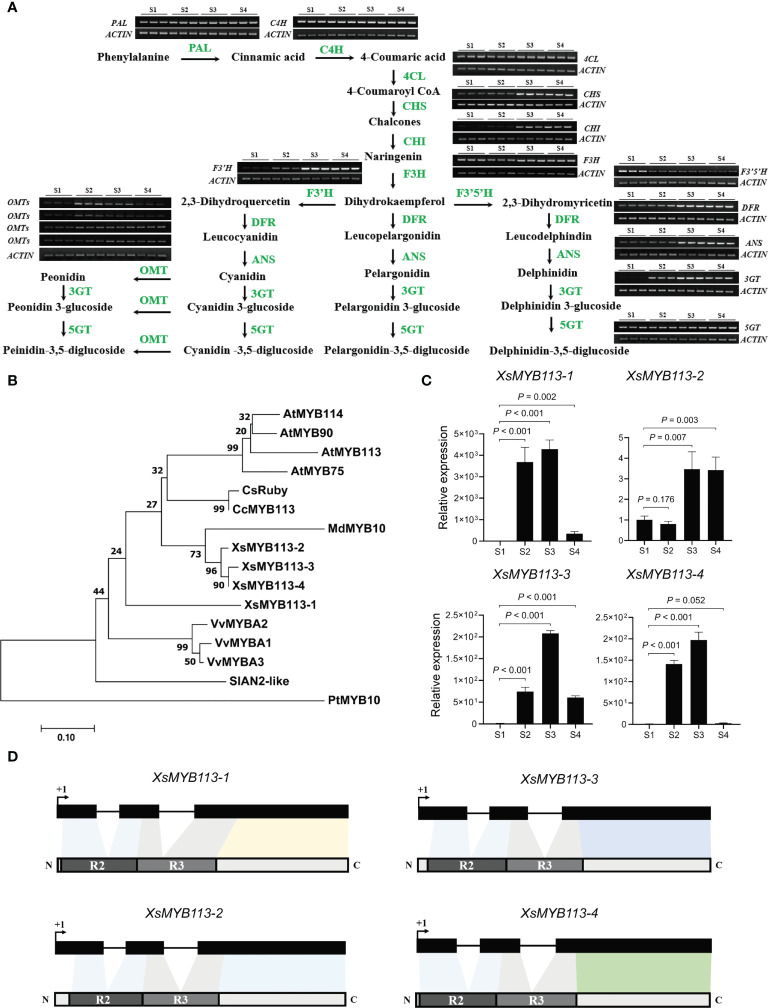
Anthocyanin-related gene expression analysis in the process of yellowhorn flower color change. **(A)** The flavonoid biosynthetic pathway relevant to flower color change in *X. sorbifolium*. RT-PCR analysis of expression profiles of the ABP structural genes during flower color change at S1 to S4. PAL, phenylalanine ammonia lyase; C4H, cinnamic acid 4-hydroxylase; 4CL, 4-coumarate Co-A ligase; CHS, chalcone synthase; CHI, chalcone isomerase; F3H, flavanone 3-hydroxylase; F3′H, flavonoid 3′-hydroxylase; F3′5′H, flavonoid 3′,5′-hydroxylase; DFR, dihydroflavonol 4-reductase; ANS, anthocyanidin synthase; 3GT, UDP-glucose:anthocyanidin 3-*O*-glucosyltransferase; 5GT, UDP-glucose:anthocyanidin 5-*O*-glucosyltransferase; OMT, anthocyanin *O*-methyltransferase. The samples at each developmental stage were repeated for 3 times. ABP, anthocyanin biosynthesis pathway. **(B)** The phylogenetic tree of the anthocyanin-related *R2R3-MYB* genes have been reported in different species. *Arabidopsis thaliana, Vitis vinifera, Citrus clementina, Populus trichocarpa, Malus domestica, Citrus sinensis, Solanum lycopersicum* and *X. sorbifolium.* The tree was constructed using Maximum Likelihood method by MEGA 7 with 1,000 bootstrap replicates. **(C)** qRT-PCR was used to detect the transcript abundances of *XsMYB113-1, XsMYB113-2, XsMYB113-3*, and *XsMYB113-4* during flower development stages S1 to S4. Data represent means ± SD of three biological replicates. *P* values were calculated based on two-sided *t* test. **(D)** Schematic diagrams of gene and protein structure of four *XsMYB113* genes in *X. sorbifolium*. The dark boxes represent the exons of four *XsMYB113* genes and solid lines represent introns of four genes. The dark gray and light gray boxes represent R2 domain and R3 domain respectively. +1 indicates the translation start site.

The anthocyanin biosynthetic pathway (ABP) is highly conserved and has been well studied in plants (Holton and Cornish, 1995). To further explore the molecular mechanisms of anthocyanin biosynthesis in yellowhorn flowers, we used RT-PCR analysis to illustrate the expression profiles of ABP genes from S1 through S4. The transcript abundances of the *CHS*, *CHI*, *F3'H*, *DFR*, *ANS*, *OMT*, and *3GT* genes in this pathway were low at S1 and S2, but increased significantly at S3, and became stable at higher abundances during S4. While the transcript abundances of some genes hardly changed, only the abundance of the *XsF3'5'H* gene transcript gradually decreased from S1 through S4 (**Figure 2A**). The timing of expression of these ABP genes is roughly consistent with the accumulation of anthocyanins in *X. sorbifolium* flowers. Thus, the observed changes in the transcript abundance of genes in the ABP could affect the synthesis of floral anthocyanins during the changes in petal color of *X. sorbifolium* flowers. The functions of three genes in the ABP (*XsF3’H*, *XsOMT*, and *XsF3’5’H*)are particularly important during anthocyanidin accumulation. In flower tissues, the transcript abundance of *XsF3'H* increased while that of *XsF3'5'H* decreased from S1 through S4. This expression pattern could have contributed to the greatly increased content of cyanidin and its derivatives observed in yellowhorn flowers. Accumulation of cyanidin-3,5-diglucoside was associated with significant increases in *Xs3GT* and *Xs5GT* transcript abundances at S3 and S4. Finally, the accumulation of peonidin and its derivatives correlated with the high transcript abundances of the *XsOMT* and *Xs3GT* genes at S3 (**Figure 2A**). The increased expression of ABP genes during changes in *X. sorbifolium* flower color were consistent with our metabolomics showing that anthocyanins accumulate to their highest levels in flowers at S4.

The above results indicated that the anthocyanin accumulation is related to the anthocyanin biosynthesis pathway, and the change of flower color is highly related to the expression pattern of some key genes in the pathway during yellowhorn flower development.

### Four R2R3-MYB transcription factors may regulate anthocyanin biosynthesis during flower color change

By analyzing the expression levels of each gene in the anthocyanin biosynthesis pathway, we found that the changes in the transcription levels of these genes were mostly consistent, which seemed to be co-regulated by a certain factor. Many studies have reported that R2R3-MYB transcription factors are a large class of factors that can regulate anthocyanin synthesis by directly targeting the expression levels of anthocyanin synthesis structural genes in plants. MYB transcription *factors* (TFs) that regulate the biosynthesis of anthocyanins in *Arabidopsis* include *AtMYB75*, *AtMYB90*, *AtMYB113*, and *AtMYB114* (Stracke et al., 2001; Gonzalez et al., 2008; Dubos et al., 2010). Among these, *AtMYB90*, *AtMYB113*, and *AtMYB114* comprise a gene cluster on *Arabidopsis* chromosome 1. In cotton, MYB113 directly targets the promoter of the anthocyanin synthesis genes to regulate the petal spot development (Abid et al., 2022).

In order to explore whether the change of flower color is related to the R2R3-MYB transcription factor, we analyzed the expression pattern of the *MYB* genes in yellowhorn. To identify homologs of the *MYB113* gene in *X. sorbifolium*, we used MYB protein sequences from several species including *Arabidopsis, V. vinifera* (grape), *C. clementina* (clementine), *P. trichocarpa* (black cottonwood*)*, *M. domestica* (apple), *C. sinensis* (sweet orange), *and S. lycopersicum* (tomato) to generate a phylogenetic tree. We identified four homologous MYB TFs in *X. sorbifolium* (**Figure 2B**; **Supplementary Table S2**). The four MYB TFs in *X. sorbifolium* were most similar to *AtMYB113*, so we named them *XsMYB113-1* (EVM0022315), *XsMYB113-2* (EVM0000778), *XsMYB113-3* (EVM0004297*)* and *XsMYB113-4* (EVM0021961).

We used qRT-PCR to detect the expression of genes encoding these four *X. sorbifolium* MYB TFs and found that their transcript abundance gradually increased during the process of flower color change in *X. sorbifolium*. Transcript abundance of the genes was low in S1, began to increase at S2, and reached their maxima at S3, but decreased again at S4 (**Figure 2C**). qRT-PCR results showed that the expression patterns of the four *XsMYB113* genes were consistent with those of anthocyanin synthesis structural genes (**Figure 2A**). Combined with the function of *MYB113* gene in other species that can positively regulate the synthesis of anthocyanins, we hypothesized that these *XsMYB113* genes are likely to be involved in the synthesis of anthocyanins in the process of yellowhorn flower color change. Further analysis found that, the four MYB TFs belong to the R2R3-MYB TF family and have complete R2 and R3 domains, indicating that their potential functions are complete (**Figure 2D**; **Supplementary Figure S2**).

*MYB113* encodes an R2R3-MYB TF that can interact with other molecules to positively regulate ABP genes in various plant species. The target genes downstream of *MYB113* include *F3'H* and *DFR* (Liu et al., 2016; Li et al., 2017). These results show that four MYB TFs may be involved in anthocyanin biosynthesis during flower petal color change in *X. sorbifolium*.

### Overexpression of *XsMYB113-1* can strongly activate anthocyanin accumulation in tobacco

**Figure 3:**
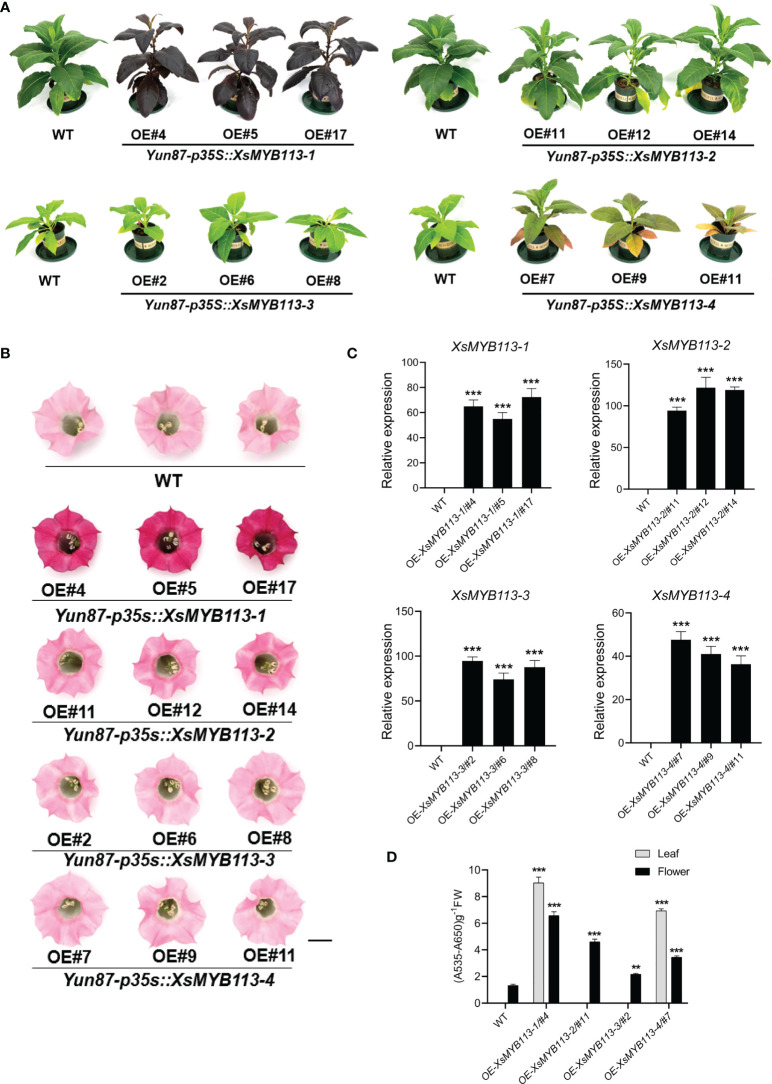
Functional analysis of *XsMYB113* genes over expressed in transgenic tobacco. Seedling **(A)** and flower color **(B)** phenotypes of *XsMYB113* gene overexpression in tobacco. The experiment was repeated independently three times with similar results obtained each time. Bar = 10 cm and 1 cm in **(A)** and **(B)**, respectively. OE represents overexpression. Yun87 represents wild type tobacco. p35S represents the promoter of CaMV 35S. **(C)** qRT-PCR was used to detect transcript abundances of *XsMYB113* genes overexpressed in transgenic tobacco relative to that of *NtEF1-α*. Data represent means ± SD of three biological replicates. Experiments were performed with three independent technical replicates and three biological samples. The triple asterisk indicates that the gene expression level in transgenic lines are significantly different from that compared with wild type according to two-sided *t* test (*P* < 0.001). **(D)** Determination of total anthocyanin contents in leaves and flowers of wild-type and transgenic tobacco plants overexpressing *XsMYB113*. These experiments were repeated three times for each independent line. The double asterisk and triple asterisk indicate that the anthocyanins content in transgenic lines are significantly different from that compared with wild type, *P* < 0.01 and *P* < 0.001, respectively, according to two-sided *t* test.

To further examine the functions of *XsMYB113-1*, *XsMYB113-2*, *XsMYB113-3*, and *XsMYB113-4*, we generated stably transformed lines of tobacco overexpressing these genes under the control of the cauliflower mosaic virus (CaMV) 35S promoter. The constructs *35S::XsMYB113-1*, *35S::XsMYB113-2*, *35S::XsMYB113-3*,and *35S::XsMYB113-4* were created and transformed into wild-type (WT) tobacco (*Nicotiana tabacum*) leaf discs (Horsch et al., 1985) and plants were regenerated. We assayed the transgenic plants using genomic PCR using primers specific to the four *MYB113* sequences. For each construct, three independent transgenic lines were selected. Overexpression from *35S::XsMYB113-1* resulted in accumulation of purple-red anthocyanin in the entire tobacco plant compared with those overexpressing *XsMYB113-2*, *XsMYB113-3*, or *XsMYB113-4* (**Figure 3A**). The flower color of *XsMYB113-*1-overexpressing plants directly became purple compared to wild type (**Figure 3B**). The transgenic *35S::XsMYB113-1* or *35S::XsMYB113-4* tobacco plants overexpressing *XsMYB113-1* or *XsMYB113-4*, respectively,showed anthocyanin accumulation in their leaves. The transgenic *35S*::*XsMYB113-2* or *35S*::*XsMYB113-3* tobacco plants overexpressing *XsMYB113-2* or *XsMYB113-3*, respectively, both exhibited green coloration over the entire plant, the same color as the wild-type tobacco. The *35S::XsMYB113-2*, *35S::XsMYB113-3*,and *35S::XsMYB113-4* transgenic plants showed small amounts of anthocyanin accumulation in the flowers compared to *35S::XsMYB113-1* (**Figure 3D**; **Supplementary Figure S3**). We analyzed the transgenic plants using qRT-PCR to determine the individual transcript abundances of *XsMYB113-1*, *XsMYB113-2*, *XsMYB113-3*, and *XsMYB113-4* (**Figure 3C**).

The expression of the endogenous *NtAN2* gene, which encodes a MYB TF that controls anthocyanin production in tobacco (Huang et al., 2012), was also analyzed in the transgenic lines. Overexpression of *XsMYB113*s did not induce expression of *NtAN2* in transgenic tobacco (not detectable by qRT-PCR; data not shown). Overexpression of the exogenous *XsMYB113* genes in tobacco did not affect the expression of the endogenous *NtAN2* tobacco gene, which suggests that *XsMYB113* genes and *NtAN2* had no homologous gene interaction.

The above results indicated that the overexpression of exogenous *XsMYB113-1* gene, rather than other three genes, resulted in a strong phenotype in tobacco. *XsMYB113-1* might represent a key TF-encoding gene involved in the regulation of anthocyanin synthesis in *X. sorbifolium*.

### DNA methylation on transposon upstream of *XsMYB113-1* correlates with its transcription

**Figure 4:**
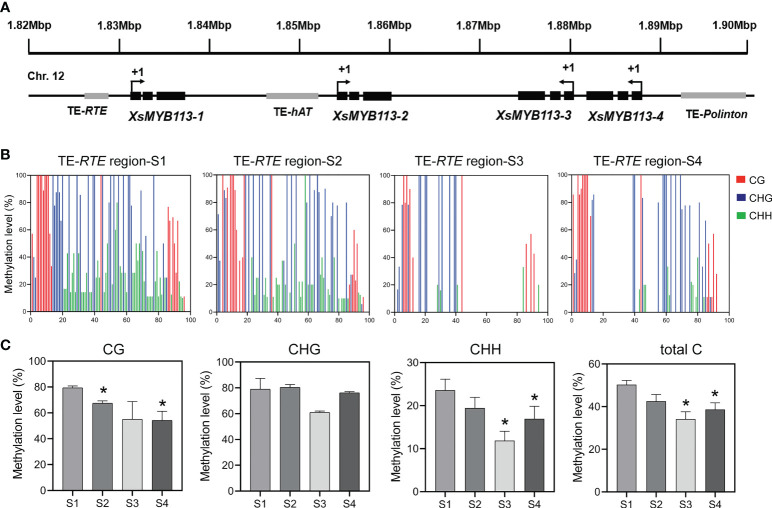
Methylation status of TE-*RTE* region near *XsMYB113-1* gene. **(A)** Schematic diagram of a gene cluster formed by four *XsMYB113* genes and nearby transposons. The dark boxes represent exons, the grey boxes indicate transposons. +1 indicates the translation start site. TE, transposable element. The black arrow indicates the transcription direction of genes. **(B)** Cytosine methylation levels of *RTE* transposon upstream of *XsMYB113-1* estimated using BSP-PCR. The *x* axis represents the cytosine position of the annotated transposon (from 5 ‘to 3’) upstream of *XsMYB113-1* gene in the yellowhorn genome, setting the position of the first cytosine at the 5 ‘end of the annotated transposon to 1. **(C)** Methylation levels of different types of cytosine in the *RTE* transposon region upstream of *XsMYB113-1* gene. *P* values were calculated based on two-sided *t* test (*P < 0.05). 500 ng genomic DNA was extracted from four different developmental stages of yellowhorn flowers for bisulfite modification, and the product was used as a template to amplify the *RTE* transposon sequence. 30 independent clones of PCR were sequenced, and the data were analyzed by Kismeth software. The experiment was repeated 3 times independently.

The expression of *XsMYB113-1* gradually changed with the development of flower, and played an important role in the process of yellowhorn flower color change. Next, we explored the reasons for the change of *XsMYB113-1* gene expression. First, we focused on the distribution of the four *XsMYB113* genes in chromosomes and found that, these four MYB TFs cluster on *X. sorbifolium* chromosome 12 together with transposons (**Figure 4A**). Repbase (Kohany et al., 2006) was used to identify transposon sequences near the *XsMYB113* genes. The promoter region of *XsMYB113-1* gene has an *RTE*-type non-LTR transposon of about 350 bp. A *hAT*-type DNA transposon of about 2.6 kb is located in the promoter region of the *XsMYB113-2* gene. Another *Polinton*-type DNA transposon with a length of 440 bp lies in the promoter region of the *XsMYB113-4* gene (**Figure 4A**; **Supplementary Table S3**).

Considering that the transcript abundance of *XsMYB113-1* gene increase sharply at S3 (**Figure 2C**) and the transposon was distributed in the promoter region of *XsMYB113-1* gene (**Figure 4A**), we proposed DNA methylation may cause the expression change of *XsMYB113-1* gene. Then, we focused on analyzing DNA methylation levels upstream of *XsMYB113-1*. To test this hypothesis, we analyzed the methylation level on the *RTE*-type non-LTR transposon, upstream of *XsMYB113-1*, by bisulfite sequencing (BSP)-PCR. At S3, the level of CHH methylation was significantly diminished (*P* < 0.05) in this transposon region (**Figures 4B, C**). Total DNA methylation and CG methylation exhibited a downward trend, but CHG methylation hardly changed from S1 to S4 in this region (**Figures 4B, C**).

In conclusion, we propose that the expression of *XsMYB113-1* gene may have been affected by the methylation, mainly at CHH, of the transposon near it.

### The reduced activity of DNA methyltransferase is related to the reduction of DNA methylation level on transposons in the change of flower color

The level of DNA methylation can be regulated by the activity of DNA methyltransferase. The reduced DNA methylation level on transposons could be caused by the reduced DNA methyltransferase activity during yellowhorn flower color change. In order to verify whether the change of DNA methylation level on transposon is related to the activity of DNA methyltransferase, we detected the expression of DNA methyltransferase genes during yellowhorn flower color change. DNA methylation in CG, CHG, CHH contexts in *Arabidopsis thaliana* is catalyzed by MET1, CMT3, CMT2, and DRM2, respectively (Zhang and Zhu, 2012).

**Figure 5:**
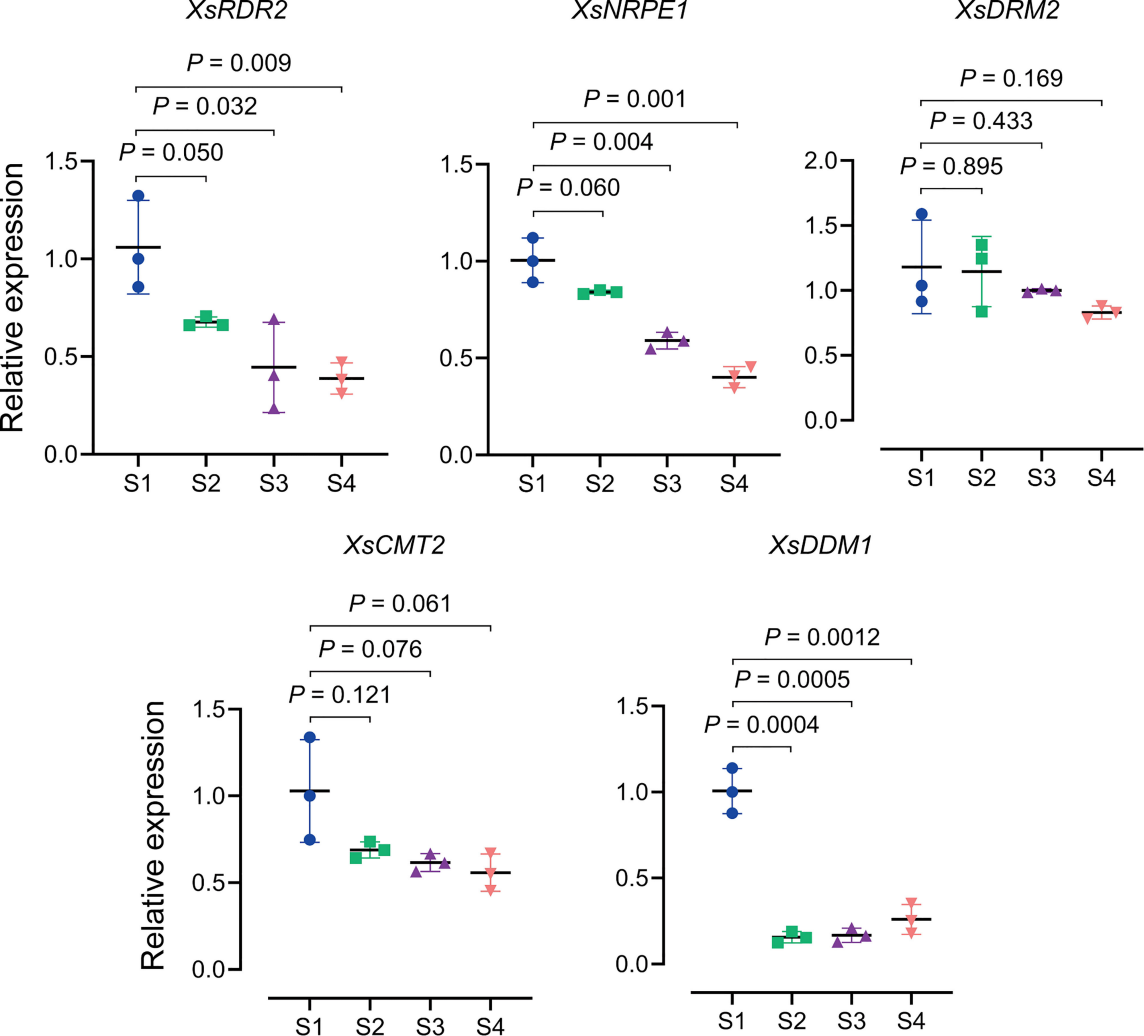
Expression patterns of DNA methylation related genes during yellowhorn flower color change. qRT-PCR was used to detect the transcriptional expression level of *XsDRM2*, *XsCMT2*, *XsDDM1*, *XsRDR2* and *XsNRPE1* from S1 to S4. We used the *XsACTIN* gene as an internal control. Data represent means ± SD of three biological replicates. Experiments were performed with three independent technical replicates and three biological samples. *P* values were calculated based on two-sided *t* test.

Using blastp, we identified DNA methyltransferase orthologs and related genes in the yellowhorn genome and constructed a phylogenetic tree (**Supplementary Figure S4**). We used qRT-PCR to estimate the transcript abundance of these genes and found that the transcript abundance of *XsRDR2*, *XsNRPE1* (a DNA directed RNA polymerase E known to determine CHH methylation), *XsDRM2*, *XsCMT2* and *XsDDM1* decreased in *X. sorbifolium* flowers from S1 through S4 (**Figure 5**). However, the transcriptional level of *XsMET1* gene was increased through S1 to S4, while the expression of *XsCMT3* gene was decreased at S2 and increased at S3 (**Supplementary Figure S5**). Thus, the observed decrease in transcript abundance of *XsRDR2*, *XsNRPE1*, *XsDRM2*, *XsCMT2* and *XsDDM1* genes was consistent with the observed decreased DNA methylation level on transposon upstream of *XsMYB113-1* gene. In conclusion, we propose that the reduced DNA methylation level on the transposon near *XsMYB113-1* gene may be due to the decreased expression of these DNA methyltransferase genes during yellowhorn flower color change.

In summary, DNA hypomethylation on transposon near *XsMYB113-1* was found to upregulate MYB gene expression level. Decreased expression of DNA methyltransferase genes might affect the methylation states of transposon near the *XsMYB113-1* gene and further influence gene expression.

## Discussion

### Accumulation of anthocyanin is associated with increased transcript abundance of ABP structural genes during yellowhorn flower color change

The phenomenon of gradual change of color during the flowering process that is uncommon in nature. Changes in the color of the petals of yellowhorn flowers are associated with increased expression of genes in the ABP and the consequent accumulation of cyanidin and other anthocyanins (**Figure 1A**). The accumulation of cyanidin, peonidin, and their derivatives were consistent with the increases in the transcript abundances of the ABP structural genes *XsF3'H, XsDFR, XsANS, Xs3GT, Xs5GT*, and *XsOMT* involved in their biosynthesis (**Figure 2A**). When flowers have just opened at S2, the inner base of the petals are yellow. This could be due to the accumulation of carotenoids and we did not detect carotenoids in flowers at that stage. At S2, the transcript abundance of most of the ABP structural genes was not high and anthocyanins had not yet accumulated (**Figure 2A**). The color of the inner base of petals changes from yellow to orange at S3, possibly due to the increased transcription of the ABP structural genes and the accumulation of anthocyanins. At the S4 development stage, the inner base of the petals has turned completely purple and the cyanidin and other anthocyanins have reached their highest levels.

The expression patterns of the two key genes *XsF3'H* and *XsF3'5'H* in the anthocyanin biosynthesis pathway play an important role during yellowhorn flower color change. The reduced expression of *XsF3'5'H* gene may be due to the presence of some transcriptional inhibitors that regulate its transcription.

### *XsMYB113* gene cluster regulates anthocyanin accumulation

In *Arabidopsis* the MYB TF-encoding genes *AtMYB75, AtMYB90, AtMYB113*, and *AtMYB114* positively regulate anthocyanin biosynthesis (Stracke et al., 2001; Gonzalez et al., 2008). We noticed that *AtMYB75*, *AtMYB90, AtMYB113*, and *AtMYB114* lie in a tandem gene cluster in the *Arabidopsis* genome, but we have not seen any reports on this *MYB* gene cluster. Tomato has a cluster of four R2R3-MYB TF-encoding genes. Only one of these genes, *SlAN2-like*, positively regulates the biosynthesis of anthocyanins. *SlAN2-like* is highly expressed in light-exposed, anthocyanin-pigmented tomato skin. The other three genes are barely expressed in shaded or light-exposed tomato fruit skin. SlAN2-like may be a master regulator of anthocyanin biosynthesis (Sun et al., 2020). In the Chinese box orange genome, the MYB regulatory TF-encoding genes *Ruby1* and *Ruby2* also form a gene cluster and their encoded proteins positively regulate the biosynthesis of anthocyanins. The truncated protein encoded by *Ruby2^short^*, a mutant of *Ruby2*, functions as an inhibitor of anthocyanin biosynthesis. *Ruby2^short^* and *Ruby1* regulate the biosynthesis of anthocyanins in oranges by competing for their common bHLH (basic helix-loop-helix) partner to form a regulatory complex (Huang et al., 2018).

We identified a gene cluster containing four R2R3-MYB-encoding genes in yellowhorn (**Figure 4A**; **Supplementary Figure S2**). These four R2R3-MYBs were closely related to the R2R3-MYB factors known to regulate anthocyanin biosynthesis in other species (**Figure 2B**). These MYBs have complete R2 and R3 conserved domains, indicating that their functions might be redundant (**Figure 2D**; **Supplementary Figure S2**). The transcript abundance of these four R2R3-MYB genes was significantly increased during anthocyanin accumulation (**Figures 2B, C**), indicating that they could all be involved in anthocyanin biosynthesis. Heterologous expression of these four R2R3-MYB TF genes in transgenic tobacco showed that each of the four lines overexpressing *XsMYB113* genes accumulated different levels of anthocyanin compared to WT tobacco. *XsMYB113-1* likely plays a major role, while *XsMYB113-2*, *XsMYB113-3*, and *XsMYB113-4* may have some roles in the regulation of anthocyanin accumulation in these transgenic lines. Overexpression of the exogenous *XsMYB113-1* gene in tobacco did not affect the expression of the endogenous *NtAN2* tobacco gene, which suggests that *XsMYB113-1* gene mediated anthocyanin synthesis in tobacco not by activating endogenous *NtAN2* gene expression, but because of its strong ability to activate anthocyanin synthesis.

Tobacco lines overexpressing *XsMYB113-2* and *XsMYB113-3* only accumulate anthocyanins in flowers, which suggests that expression of these two genes might be tissue-specific (**Figure 3B**). The functions of these MYBs in transgenic tobacco differ from those in tomato, where four *R2R3-MYB* genes form a gene cluster but only *SlAN2-like* is expressed during light-induced anthocyanin biosynthesis, while the other three *R2R3-MYB* genes in the cluster may not participate in this process (Sun et al., 2020).

### Epigenetic regulation of the *XsMYB113-1* gene

During flower color change in *X. sorbifolium*, we propose that the decrease in DNA methylation of the transposon upstream of the *XsMYB113-1* gene (**Figures 4B, C**), resulting in an increase in the abundance of *XsMYB113-1* transcripts.

Another important factor for maintenance of methylation states, DDM1, a member of the *Snf2* family, is a nucleosome remodeler necessary for DNA methylation (Jeddeloh et al., 1999; Lippman et al., 2004). The SNF2 chromatin remodeling factor moves along the DNA, hydrolyzing ATP, and changes the composition and position of the nucleosomes to allow other proteins to enter regions of the DNA (Ryan and Owen-Hughes, 2011). In *Arabidopsis ddm1* loss-of-function mutants, DNA methylation of repetitive sequences is greatly reduced and the TEs in these regions become strongly transcriptionally activated (Lippman et al., 2004). As DRM2 is responsible for the CHH methylation of RdDM, CMT2 can mediate DDM1-dependent CHH methylation without requiring siRNA targeting of those DNA regions. DNA methylation within heterochromatic regions containing long TEs can require DDM1 in addition to MET1 (CG), CMT3 (CHG), and CMT2 (CHH). In contrast, methylation within short TEs within euchromatic regions is controlled mainly by RdDM *via* DRM2 (Zemach et al., 2013). Therefore, we also examined the expression patterns of *XsDDM1* and *XsCMT2* in *X. sorbifolium*. The relative abundance of transcripts from *XsDDM1* and *XsCMT2* gradually decreased during flower color change in *X. sorbifolium* from S1 through S4 (**Figure 5**). We could not confirm whether the *XsMYB113* gene cluster region was located in a euchromatic or heterochromatic region of the *X. sorbifolium* genome, so we propose that XsDDM1 and XsCMT2 might also participate in the process of flower color change. In summary, we propose that CHH methylation on transposon near the *XsMYB113-1* might be cooperatively regulated by XsDDM1, XsCMT2, and XsDRM2 to control the expression of the *XsMYB113-1* gene.

Dynamic DNA methylation can also influence other plant developmental processes (He et al., 2009; Ning et al., 2020). The ripening of sweet orange fruit is accompanied by global DNA hypermethylation, while the ripening of tomato fruit is accompanied by global DNA hypomethylation, indicating additional roles for the DNA methylation in plant development (Liu et al., 2015; Lang et al., 2017; Huang et al., 2019). The expression levels of several DNA methylation maintenance related genes, including *XsDDM1*, *XsCMT2*, and *XsCMT3* were reduced during yellowhorn flower development. The reduction of these genes is likely to be important for genome-wide DNA demethylation, which is passive DNA demethylation.

The recent publication of *X. sorbifolium* genome assemblies will help us to better understand the development of flowering in this and its related species. These genome assemblies have revealed important characteristics of the *X. sorbifolium* genome such as its high content of repeat sequences (>50%) and relatively low heterozygosity (0.75%) (Bi et al., 2019). The available genome sequences will inform subsequent molecular breeding of *X. sorbifolium*.

### Model for yellowhorn flower color change

**Figure 6:**
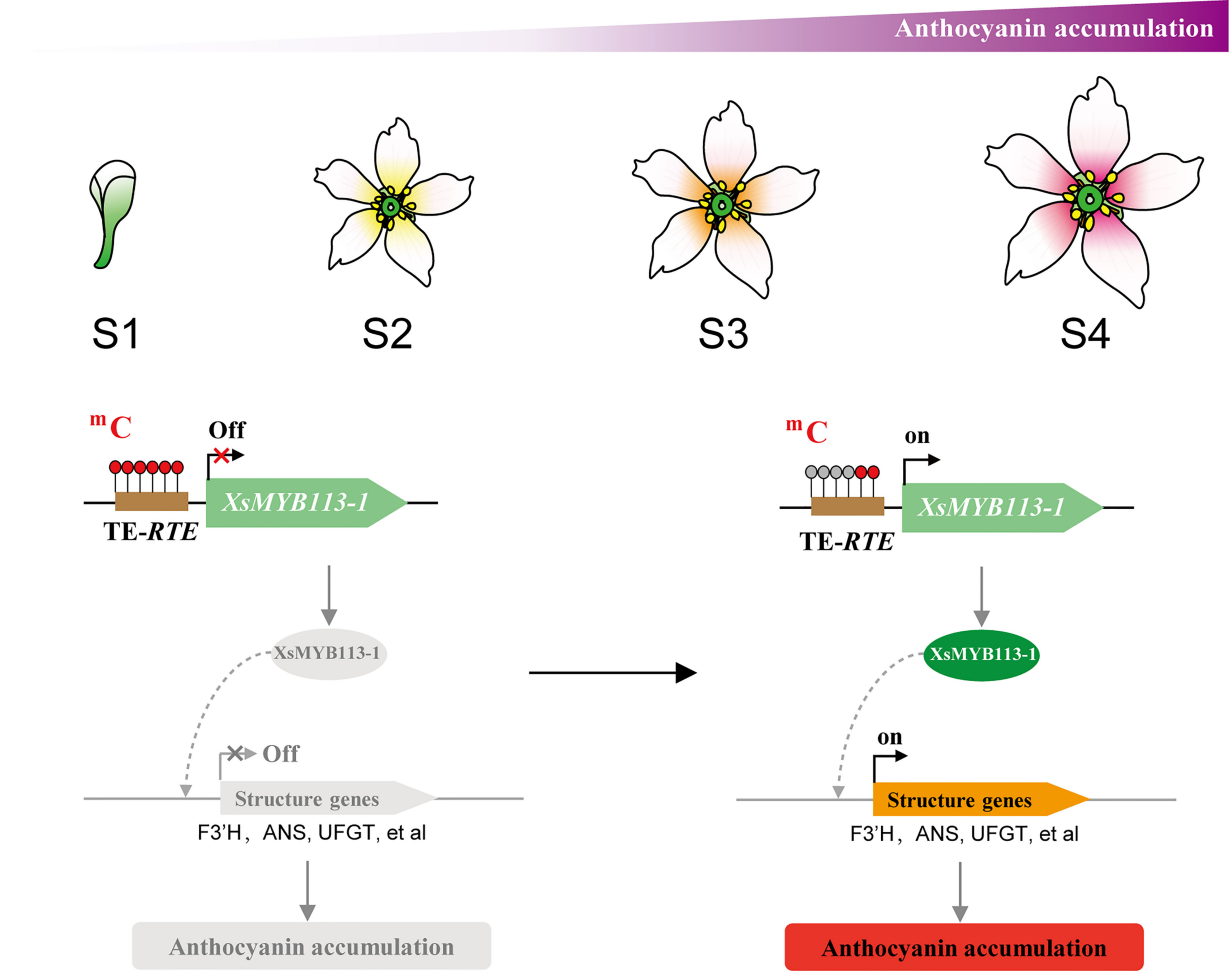
Model for anthocyanin accumulation and *XsMYB113-1* regulated by DNA methylation causing changes in anthocyanin biosynthesis during flower color change in *X. sorbifolium*. The flower schematic diagram from S1 to S4 represents yellowhorn four different developmental flower stages with different colors at the inner base of petals. The brown box represents transposon element. The red filled circle on the transposon represents the methylated cytosine, and the gray filled circle represents the unmethylated cytosine. The black arrow on the gene represents the transcription starting site and transcription direction.

Our data suggest a model for flower color change in *X. sorbifolium.* Before yellowhorn flowers open, the high DNA methylation level on transposon upstream of *XsMYB113-1* gene inhibits the expression of *XsMYB113-1* gene, thereby inhibiting the activation of downstream anthocyanin biosynthetic genes and thus the accumulation of anthocyanins. Upon opening of the flowers, DNA methylation on the TE near the *XsMYB113-1* gene decreases, and the expression of *XsMYB113-1* gene increases, leading to activation of the downstream anthocyanin biosynthetic genes and accumulation of anthocyanins. With the gradual accumulation of purple anthocyanins, the color of the inner base of the yellowhorn petals gradually turns purple (**Figure 6**).

Our ability to conduct transgenic analysis of gene function in yellowhorn is currently limited due to the long maturation cycle of this species, thus limiting studies of flower color and whole flower development. However, addressing the urgent need for rapid and effective transgenic methods to confirm the results of this study would promote studies of epigenetic regulation in yellowhorn and other woody plants. Although a genetic transformation system of yellowhorn has not yet been completely established, the further research of yellowhorn will be highly dependent on the development of this technology. Future research on epigenetic inheritance of yellowhorn will need an efficient transgenic system.

## Data availability statement

The original contributions presented in the study are included in the article/**Supplementary Material**. Further inquiries can be directed to the corresponding author.

## Author contributions

ZZ designed the research, analyzed data, and wrote the manuscript. YL performed experiments, analyzed data, and wrote the manuscript. HW collected materials and wrote the manuscript. ZL and TZ performed bioinformatics analysis. ZJL participated in photographing yellowhorn flower color changes. LC and SW participated in experiments. YYL and SY collected and planted the plant materials. QZ participated in the analysis and discussion of article, and provided guidance and suggestions. All authors contributed to the article and approved the submitted version.

## Funding

This work was supported by grants from the Special Foundation for National Science and Technology Basic Research Program of China (2019FY100802), Innovation Project of the State Key Laboratory of Tree Genetics and Breeding (2019A02), Innovation Project of the State Key Laboratory of Tree Genetics and Breeding (2022B01), and Inner Mongolia Autonomous Region Major Science and Technology Project (ZDZX2018056).

## Acknowledgments

We would like to thank Jingpeng Sun for maintaining the plants in the glasshouse and the Yunnan Academy of Tobacco Agricultural Sciences for kindly providing seeds of the tobacco line Yun87. We would also like to thank Libing Wang, Quanxin Bi, Shouke Li, Xiaoqin Xiang, Yali Wang, Yindoleng Sai, and the Kundu Economic Forest Farm for kindly providing *X. sorbifolium* materials.

## Conflict of interest

The authors declare that the research was conducted in the absence of any commercial or financial relationships that could be construed as a potential conflict of interest.

## Publisher’s note

All claims expressed in this article are solely those of the authors and do not necessarily represent those of their affiliated organizations, or those of the publisher, the editors and the reviewers. Any product that may be evaluated in this article, or claim that may be made by its manufacturer, is not guaranteed or endorsed by the publisher.
