# Supplementary material for: A naturally-occurring phenomenon of flower color change during flower development in Xanthoceras sorbifolium
Source: Front Plant Sci. 2022 Nov 15;13:1072185. doi: 10.3389/fpls.2022.1072185 (PMC9706096; doi:10.3389/fpls.2022.1072185)
Supplement: Supplementary file 1 [file DataSheet_1.docx]

Supplementary Material

# 1 Supplementary Data

**Supplementary Figure S1.** Identification and quantitative analysis of 18 non-anthocyanidins detected by targeted metabolism during the yellowhorn flower color change.

**Supplementary Figure S2.** Alignment diagram of the amino acid sequence of four XsMYB113 proteins. The red box represents the R2 domain, and the green box represents the R3 domain.

**Supplementary Figure S3.** The picture shows the different colors of different tobacco transgenic lines after extracting anthocyanins.

**Supplementary Figure S4.** The phylogenetic tree analysis of *MET1*, *CMT3*, *CMT2*, *DRM2*, *RDR2*, *DDM1*, and *NRPE1* genes in *Arabidopsis thaliana* and *X. sorbifolium.*

**Supplementary Figure S5.** qRT-PCR was used to detect the transcriptional expression level of *XsMET1* and *XsCMT3* from S1 to S4.

**Supplementary Table S1** Basic information of *MET1*, *CMT2, CMT3, DDM1, NRPE1, RDR2* and *DRM2* genes in *Arabidopsis thaliana and X. sorbifolium*

**Supplementary Table S2** Basic information of anthocyanin-related *R2R3-MYB* genes in various plants

**Supplementary Table S3** Transposon annotation information near the *XsMYB113* gene cluster

**Supplementary Table S4** Primers used in this study

# Supplementary Figures and Tables

## 2.1 Supplementary Figures


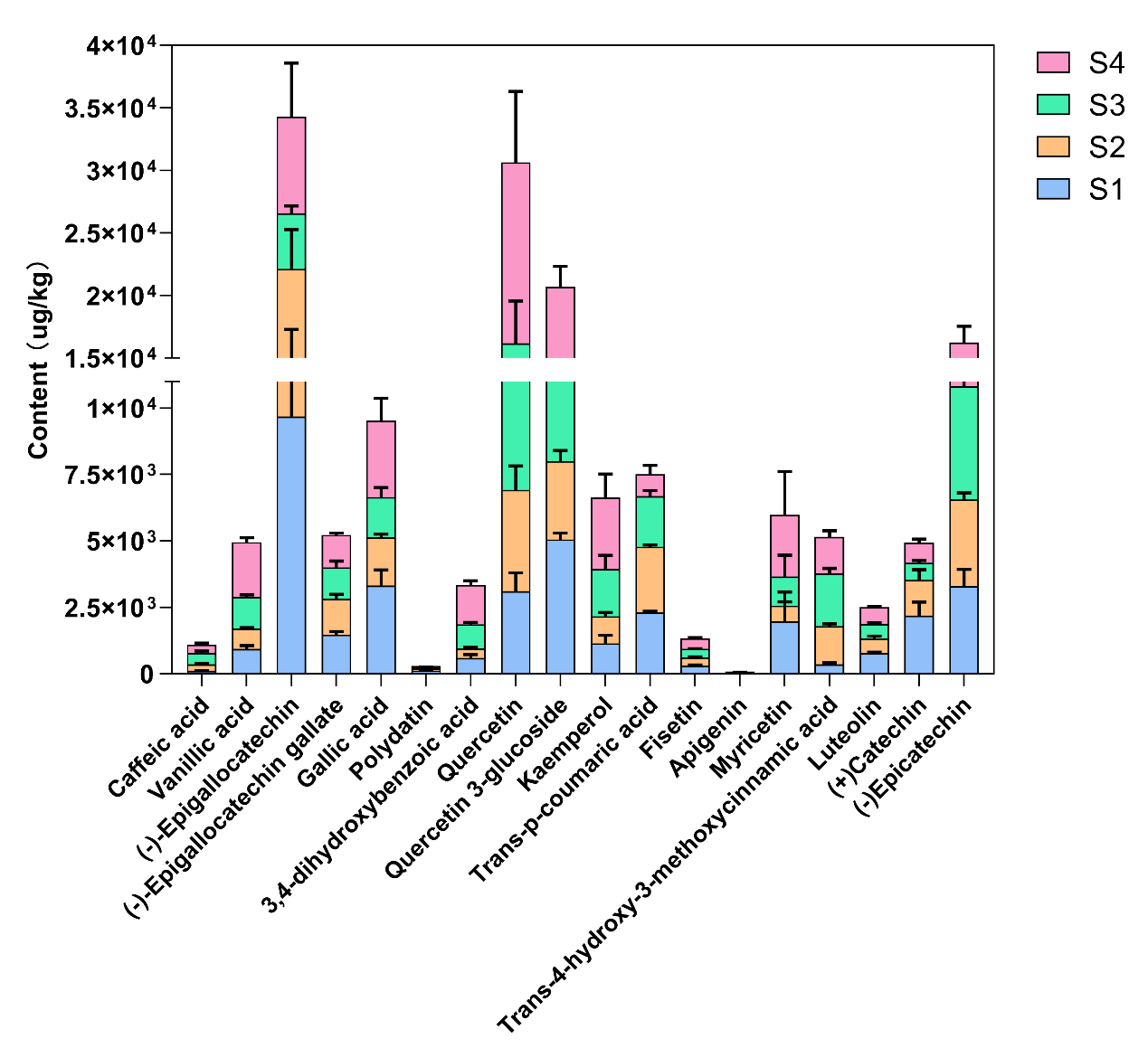


**Supplemental Figure S1.** Identification and quantitative analysis of 18 non-anthocyanidins detected by targeted metabolism during the yellowhorn flower color change. The data are the mean ± SD from three biological replicates. SD, standard deviation.


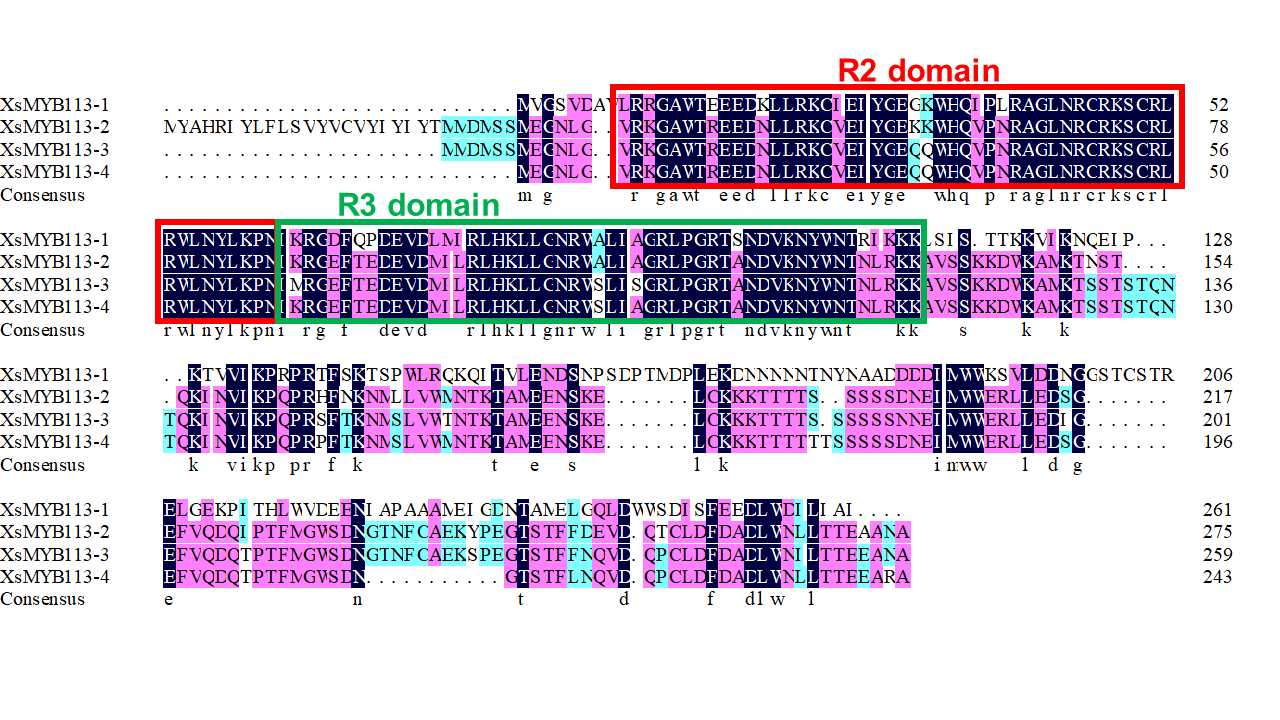


**Supplemental Figure S2.** Alignment diagram of the amino acid sequence of four XsMYB113 proteins in *X. sorbifolium*. The red box represents the R2 domain, and the green box represents the R3 domain.


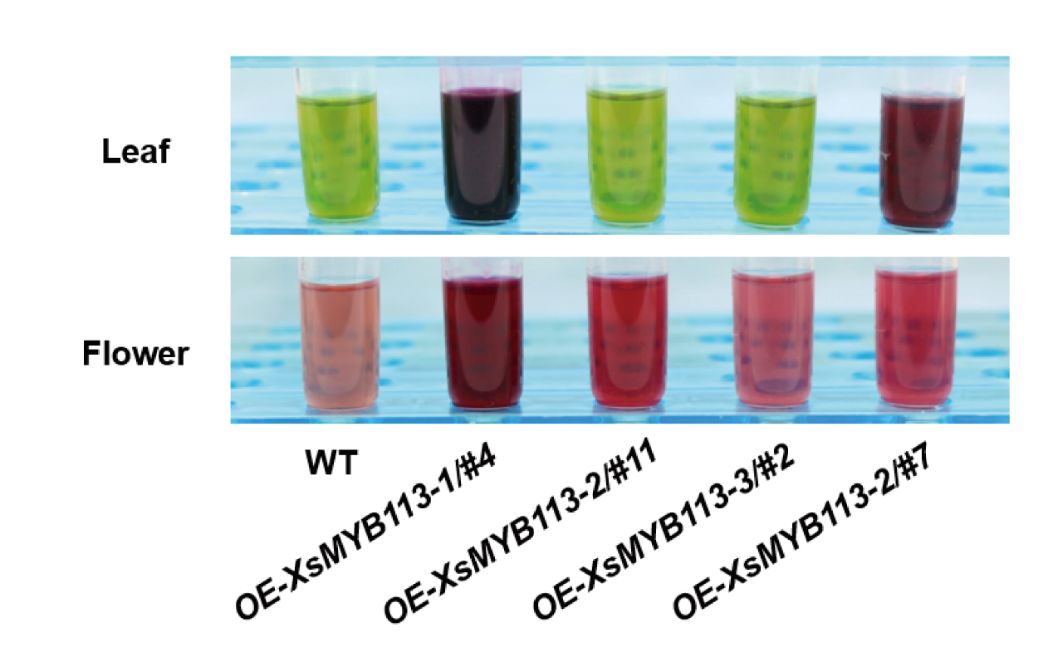


**Supplemental Figure S3.** The picture shows the different colors of different transgenic lines after extracting anthocyanins. 1% HCl in methanol (v/v) was used to extract anthocyanins from the flowers of tobacco WT, *OE-XsMYB113-1#4*, *OE-XsMYB113-2#11*, *OE-XsMYB113-3/#2* and *OE-XsMYB113-4/#7* plants. WT, wild type; OE, overexpression.


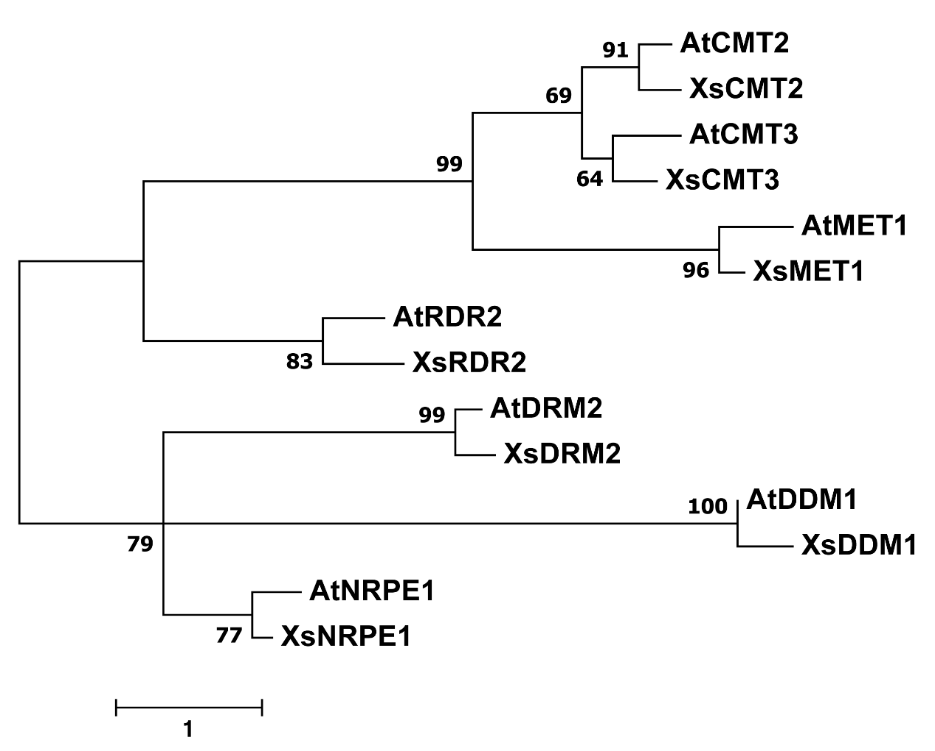


**Supplemental Figure S4.** The phylogenetic tree analysis of *MET1*, *CMT3*, *CMT2*, *DRM2*, *RDR2*, *DDM1*, and *NRPE1* genes in *Arabidopsis thaliana* and *X. sorbifolium*. The tree was constructed using Maximum Likelihood method by MEGA 7 with 1,000 bootstrap replicates.


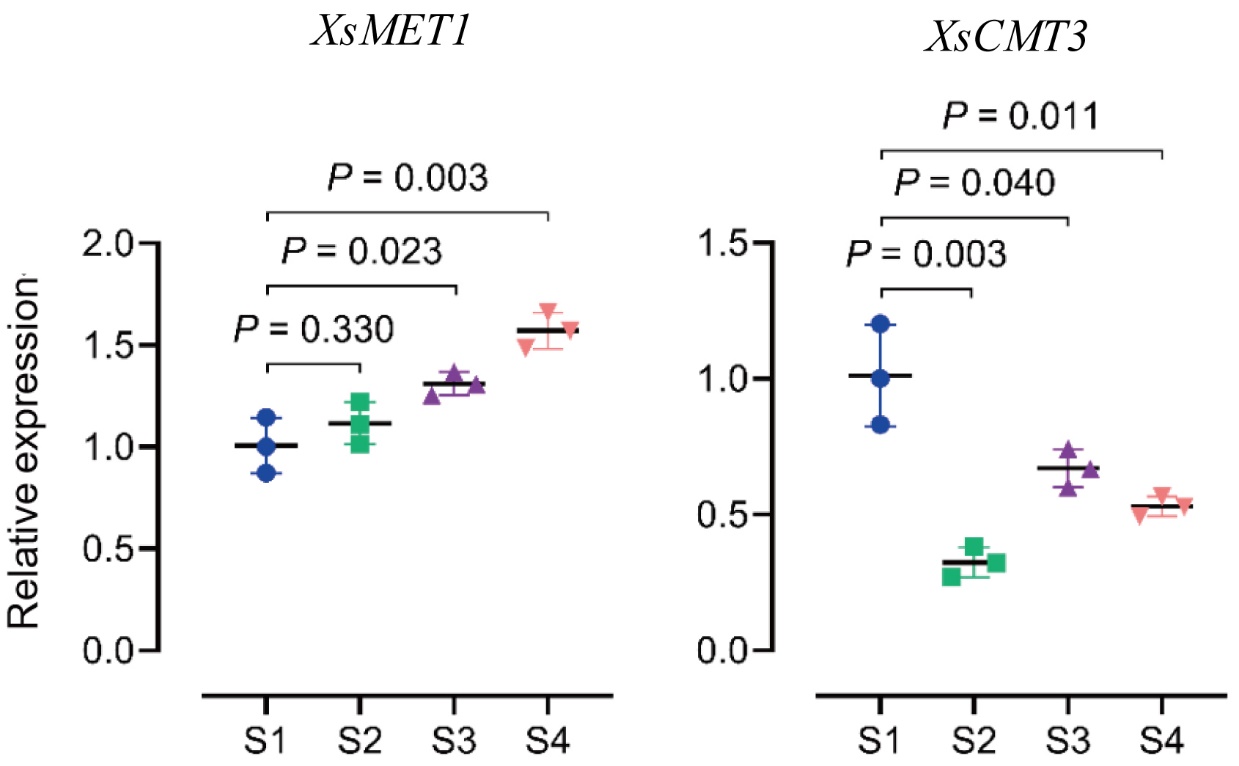


**Supplemental Figure S5.** Expression patterns of DNA methylation related genes during yellowhorn flower color change. qRT-PCR was used to detect the transcriptional expression level of *XsMET1* and *XsCMT3* from S1 to S4. We used the *XsACTIN* gene as an internal control. Data represent means ± SD of three biological replicates. Experiments were performed with three independent technical replicates and three biological samples. *P* values were calculated based on two-sided *t* test.

## Supplementary Tables

**Supplemental Table S1**. Basic information of *MET1*, *CMT2, CMT3, DDM1, NRPE1, RDR2* and *DRM2* genes in *Arabidopsis thaliana* and *X. sorbifolium*

| **Species** | **Gene** | **Gene ID** |
| --- | --- | --- |
| *Arabidopsis thaliana* | AtCMT2 | AT4G19020 |
| *Arabidopsis thaliana* | AtCMT3 | AT1G69770 |
| *Arabidopsis thaliana* | AtDDM1 | AT5G66750 |
| *Arabidopsis thaliana* | AtNRPE1 | AT2G40030 |
| *Arabidopsis thaliana* | AtRDR2 | At4G11130 |
| *Arabidopsis thaliana* | AtMET1 | AT5G49160 |
| *Arabidopsis thaliana* | AtDRM2 | AT5G14620 |
| *Xanthoceras sorbifolium* | XsCMT2 | EVM0021882 |
| *Xanthoceras sorbifolium* | XsCMT3 | EVM0020301 |
| *Xanthoceras sorbifolium* | XsDDM1 | EVM0004683 |
| *Xanthoceras sorbifolium* | XsNRPE1 | EVM0021361 |
| *Xanthoceras sorbifolium* | XsRDR2 | EVM0000836 |
| *Xanthoceras sorbifolium* | XsMET1 | EVM0004891 |
| *Xanthoceras sorbifolium* | XsDRM2 | EVM0006407 |

**Supplemental Table S2**. Basic information of anthocyanin-related *R2R3-MYB* genes in various plants

| **Species** | **Gene** | **NCBI ID** |
| --- | --- | --- |
| *Arabidopsis thaliana* | AtMYB75 | AAG42001.1 |
| *Arabidopsis thaliana* | AtMYB90 | NP_176813.1 |
| *Arabidopsis thaliana* | ATMYB113 | NP_176811.1 |
| *Arabidopsis thaliana* | ATMYB114 | AEE34502.1 |
| *Xanthoceras sorbifolium* | XsMYB113-1 | EVM0022315 |
| *Xanthoceras sorbifolium* | XsMYB113-2 | EVM0000778 |
| *Xanthoceras sorbifolium* | XsMYB113-3 | EVM0004297 |
| *Xanthoceras sorbifolium* | XsMYB113-4 | EVM0021961 |
| *Vitis vinifera (wine grape)* | VvMYBA1 | XP_010664911.1 |
| *Vitis vinifera (wine grape)* | VvMYBA2 | XP_002265048.1 |
| *Vitis vinifera (wine grape)* | VvMYBA3 | NP_001268189.1 |
| *Citrus clementina* | CcMYB113 | XP_024038103 |
| *Populus trichocarpa* | PtMYB10 | XP_002307190 |
| *Malus domestica* | MdMYB10 | ABB84753 |
| *Citrus sinensis* | CsRuby | NP_001275818.1 |
| *Solanum lycopersicum* | SlAN2-like | AUG72358.1 |

**Supplemental Table S3**. Transposon annotation information near the *XsMYB113* gene cluster

| **chromosome** | **Start** | **End** | **Class** | **Length** | **Similarity** |
| --- | --- | --- | --- | --- | --- |
| Chr12 | 1,835,074 | 1,835,431 | Non-LTR/*RTE* | 358 | 74.26% |
| Chr12 | 1,850,392 | 1,852,996 | DNA/*hAT* | 2605 | 68.73% |
| Chr12 | 1,885,706 | 1,886,145 | DNA/*Polinton* | 440 | 83.33% |

**Supplemental Table S4**. Primers used in this study

| **Purpose** | **Gene name** | **Primer sequence** |
| --- | --- | --- |
| qRT-PCR | *XsMYB113-1* | RT-MYB113-1-F: ATGGTGGGTTCTGTTGACGCAGTGCTGAGA  RT-MYB113-1-R: TTCTAGTGGATCCATGGTTGGATCGGATG |
|  | *XsMYB113-2* | RT-MYB113-2-F: GGACATGTCGTCCATGGAGGGAAATCTAGG  RT-MYB113-2-R: GCTGTTCTCTTCCATGGCGGTTTTCGTATT |
|  | *XsMYB113-3* | RT-MYB113-3-F: GGCTTCCTGGGAGAACAGCGAATGATG  RT-MYB113-3-R: TTAAATAGCATTTGCTTCTTCTGTAGTTAG |
|  | *XsMYB113-4* | RT-MYB113-4-F: GCCATGAAAACTAGTAGTACTAGTACTCAAAAC  RT-MYB113-4-R: TTAAATTGCACGTGCTTCTTCTGTAGTTAGAAG |
|  | *XsDDM1* | RT-DDM1-F: GATCTCATTGTGGCAGAATGGTCTG  RT-DDM1-R: CAATTCCTTTAGCAGTTTGCACTTCGG |
|  | *XsCMT2* | RT-CMT2-F: ATGGAATCACCAATGAAATCGTCAAACCCA  RT-CMT2-R: CCACCTTATCAATAGAGGAATGAGGGGTTC |
|  | *XsNRPE1* | RT-NRPE1-F: GGATCTTTCCATATCAATGCTTAAAAA  RT-NRPE1-R: AGGTCTTAGAAAGGTGTGCCCCTTG |
|  | *XsCMT3* | RT-CMT3-F: TTGCCCTTGCCTGGTGAGGTT  RT-CMT3-R: CTCTCAGATGGTTGGGCACCC |
|  | *XsRDR2* | RT-RDR2-F: GGTGTCGTGGCTATCGATCCG  RT-RDR2-R: GCGCGGAACGTCTGAAGCATC |
|  | *XsMET1* | RT-MET1-F: GGTTGCTGTAGGCGATTCCGT  RT-MET1-R: AGAATGCACCTCTCTCAGGCC |
|  | *XsDRM2* | RT-DRM2-F: CCAAGTTGATACGGTGGCCTACC  RT-DRM2-R: CGACTGTATCTGTTGCTGCCAGC |
|  | *XsACTIN* | RT-ACTIN-F: ACGTCACACTGGAGTGATGGTTG  RT-ACTIN-R: TGGGTTGAGAGGTGCTTCAGTAAG |
|  | *NtEF1α* | RT-NtEF1α-F: AAGCTGACTGTGCTGTCCTGA  RT-NtEF1α-R: CAACAGGGACAGTACCAATTCCACC |
| RT-PCR | *XsF3’H* | RT-F3’H-F: ATGTCTGCTCTGCTTCTCTACTCCACT  RT-F3’H-R: CGAACATGTCTATAATCATCCAAGGCCT |
|  | *XsPAL* | RT-PALs-F: TGGTGCCACTTCTCACAGACGAACCAA  RT-PALs-R: GGCGAAAATCGCTGACAGGACTTCTGATA |
|  | *XsC4H* | RT-C4Hs-F: TCATGACGGTGCCCTTCTTCACCAACAA  RT-C4Hs-R: GCAACATTGATATTCTCTACAATGTAGAG |
|  | *Xs4CL* | RT-4CLs-F: AGCGTCGCCCAACAAGTTGACGGAGA  RT-4CLs-R: GGTTCCTTTGCAAATGCCAAACACATTG |
|  | *XsCHS* | RT-CHS-F: GGTTCCGACCCAGTACCCGGCGTCGAAAAG  RT-CHS-R: CTAAGCAGCGGCAACACTGTGGAGGACAAC |
|  | *XsCHI* | RT-CHI-F: GATTGCAGTTTACTTGGAGGATGACGCCGT  RT-CHI-R: CGTCTTCGTTCAACAGTTTTGATAACCTTG |
|  | *XsDFR* | RT-DFR-F: CCACCTCTAGTTGTTGGTCCATTTCTGATG  RT-DFR-R: ATTCGAGGTAGGGATCGATTCTTTCGCGAC |
|  | *XsF3H* | RT-F3H-F: GTGGACATGGACCAAAAGGTTGTGGTCAA  RT-F3H-R: TCAAGCAAGGATCTGCTCAATAGGCTTGGC |
|  | *XsANS* | RT-ANS-F: TGACCAGGCTTCAGGCAATGTTCAAGGCTA  RT-ANS-R: GCCATTGCTGAGAATCTCGATGGTGTCTCC |
|  | *Xs3GT* | RT-3GT-F: GCCTTGTTGGTTGTCAGGATCTAGCTCACT  RT-3GT-R: CCAAGGCCTCTGCTACTGCCACAATCTCAT |
|  | *Xs5GT* | RT-5GT-F: GGCCGTTGATTCCATCTGCATTTTTGGA  RT-5GT-R: CAACTTTACTCTCACTCCTGTCTTCCAC |
|  | *XsF3’5’H* | RT-F3’5’H-F: ATGGTTCTAGACACCTTCCTCCTCAAAGAG  RT-F3’5’H-R: GCCTTTTCGGCTAGAGTCACACATACTTTG |
|  | *XsOMTs* | RT-OMTs-F: CTGGCTAGCTACTCCATCCTCACTTGCTCT |
|  |  | RT-OMTs-R: TGACATATCCACTTCATGAAAATGGCATC |
|  | *XsACTIN* | RT-ACTIN-F: AGATCCTCACTGAGAGAGGATATTCTT  RT-ACTIN-R: CCGATCTGCAATTCCAGGGAACATA |
| BSP  analysis | *RTE*-transposon fragment | BS-F: AGTTAGGAYAATGAGYAAYATTAATGTTA  BS-R: CAACCCTCCAAAATATTTRTAAACTTTATATTC |
